# Epidemiology of severe trauma in Navarra for 10 years: out-of-hospital/ in-hospital deaths and survivors

**DOI:** 10.1186/s12873-023-00818-6

**Published:** 2023-05-24

**Authors:** Eider Arbizu-Fernández, Alfredo Echarri-Sucunza, Arkaitz Galbete, Mariano Fortún-Moral, Tomas Belzunegui-Otano

**Affiliations:** 1 Emergency Department of Universitary Hospital of Navarre, Pamplona, Spain; 2 Subdirección de Urgencias de Navarra, Pamplona, Navarre, Spain; 3 Department of Statistics, Computer Science and Mathematics, Public University of Navarra, RICAPPS, Pamplona, IdiSNA Spain; 4 Emergency Department Hospital Universitario de Navarra, Pamplona, Navarre, Spain; 5 Polytrauma group, Navarrabiomed - Universitary Hospital of Navarre, Public University of Navarre, Health investigation institute of Navarre, Pamplona, Navarre, Spain

**Keywords:** Mortality, Major trauma, Epidemiology, Out-of-hospital, In-hospital, Survivors

## Abstract

**Background:**

Major trauma is a leading cause of death. Due to the difficulties to keep a registry of these cases, few studies include all subjects, because they exclude out-of-hospital deaths. The purpose of this work was to compare the epidemiological profiles of out-of-hospital deaths, in-hospital deaths, and survivors over a 10-year period (2010–2019) of patients who had been treated by Navarre´s Health Service (Spain).

**Methods:**

Retrospective longitudinal cohort study using data of patients injured by an external physical force of any intentionality and with a New Injury Severity Score above 15. Hangings, drownings, burns, and chokings were excluded. Intergroup differences of demographic and clinical variables were analysed using the Kruskal Wallis test, chi-squared test, or Fisher´s exact test.

**Results:**

Data from 2,610 patients were analysed; 624 died out-of-hospital, 439 in-hospital, and 1,547 survived. Trauma incidences remained moderately stable over the 10-year period analysed, with a slight decrease in out-of-hospital deaths and a slight increase in in-hospital deaths. Patients of the out-of-hospital deaths group were younger (50.9 years) in comparison to in-hospital deaths and survivors. Death victims were predominantly male in all study groups. Intergroup differences regarding prior comorbidities and predominant type of injury were observed.

**Conclusions:**

There are significant differences among the three study groups. More than half of the deaths occur out-of-hospital and the causative mechanisms differ in each of them. Thus, when designing strategies, preventive measures were considered for each group on a case-by-case basis.

## Background

Major trauma, a major public health issue concealed from public view, is associated with high mortality rate and impairment worldwide [1]. A patient diagnosed with major trauma has experienced an external physical force with a New Injury Severity Score (NISS) above 15 [2]. The incidence in Europe varies between 30 and 52 cases per 100,000 population, with great impact on direct and indirect economic costs [3]. Trauma registries help improve the efficiency and quality of care of these patients, allow the development of epidemiological and clinical research studies, and assess the outcomes methodically. In 2009, the WHO recommended the creation of public trauma registries [4]. In this study, we use the first population-based major trauma registry developed in Spain, the Major Trauma Registry of the autonomous community of Navarre [5] Numerous publications have been published using this registry [2, 6].

Although many major trauma patients die out-of-hospital, few studies report it and rather focus their work on in-hospital deaths (IHDs) [7]. In Spain, around one IHD occurs per every five out-of-hospital deaths (OOHDs) (1:5 ratio), while in Europe the rate lowers to 1:9^8^. The characteristics of IHDs and OOHDs after a major trauma differ [7–11]. Thus, preventive strategies should be tailored considering the setting and be actively implemented by official entities. This would help establish prevention mechanisms in the health systems, as seen for other pathologies.

The aim of this study is to characterize major trauma patients in Navarre using data collected over for 10 years, comparing the groups of patients who die in out-of-hospital, die in hospital and those who survive, identifying factors associated with mortality and to facilitate the implementation of preventive measures.

## Materials and methods

This study is a retrospective longitudinal cohort study that includes data from Navarra´s Major Trauma Registry. Our study population is the autonomous community of Navarra because our database is population based. Database inclusion criteria were patients injured by an external physical force of any intentionality collected over a period of 10 years (January 1, 2010, to December 31, 2019) and with a NISS above 15 attended by the Navarra emergency system or admitted to the Navarra´s Institute of Legal Medicine due to deaths in situ. Subjects admitted to the hospital after more than 24 h post-injury, chokings, drownings, hangings, or burns without other traumatic injuries were excluded. A web application that allowed users to collaborate in the provision of data for trauma cases was developed for the registration of patients both by the reference person of the Institute of Legal Medicine and by the.

hospital referents. The overall supervision and administration of the system was conducted by a data manager that ensured compliance with the inclusion criteria and the introduction of data for each patient. Usually, a prehospital user identifies a possible case of trauma (personal data, date, and receiving center) and prehospital information: Revised Trauma Score (RTS), score on the GCS, mechanism, and intent of the injury. Then, a hospital user diagnoses the patient and completes the patient’s records: Injury Severity Score (ISS), NISS, RTS, and previous comorbidity. Then, the data manager supervises the inclusion criteria and maintains or removes the patient from the database [12–14].

The Autonomous Community of Navarre has a land area of 10,421 square kilometres, with a population of 657,776 inhabitants according to the Spanish Statistical Office [15]. Urgent care services located in the capital of the Community include a tertiary hospital, the Navarre SOS Coordination Centre, three Advanced Life Support ambulances, and a medical helicopter. Additionally, there are two regional hospitals located in the South and East of the Community, each with one Advanced Life Support ambulance. Several Basic Life Support ambulances compose the rest of the emergency system with emergency medical technicians and rural urgent care services performed by physicians and nurses. Out-of-hospital and emergency unit physicians are mostly trained in Family and Community Medicine, as to date, the speciality Urgent Care and Emergency does not exist in the Spanish health system.

The variables included in the study followed the Utstein-style guidelines on uniform reporting [16]. We assessed demographic variables (age and sex), comorbidity (ASA-Physical Status), type of accident (blunt or penetrating), intentionality, mechanism, orotracheal intubation, oxygen therapy, fluid therapy, Injury Severity Score (ISS), NISS, TRISS and Revised Trauma Score (RTS) [17]. Table 1. The ISS calculations are the same in the forensic anatomical institute and in the hospital. The prognostic scales in case of out-of-hospital death were measured with the AIS lesions collected by the forensic doctor in the autopsies and in-hospital death with the data from the computerized clinical history.

**Table 1:** Definition of scales

ISS	Injury Severity Score	Standardizes severity of traumatic injury based on worst injury of 6 body systems
**NISS**	New Injury Severity Score	It is the sum of the square of the three lesions with the highest AIS score of the body regions, regardless of the anatomical region
**RTS**	Revised Trauma Score	Quantifies severity of trauma injuries based on GCS, blood pressure, and respiratory rate
**TRISS**	Trauma Score and Injury Severity Score	Estimates the probability of survival for trauma patients using the type of trauma, RTS, ISS and the age

Patient injuries were registered according to the 2008 version of the Abbreviated Injury Scale [1]. To document dependent variables (death or survival) a follow-up was carried out after the traumatic event using patient´s computerized medical records form Navarre´s University Hospital.

Quantitative variables are expressed as mean and standard deviation (SD) or median and interquartile range (IQR) and categorical variables as frequency and percentage. To determine intergroup differences of continuous variables the Kruskal Wallis test was applied. Comparisons of categorical variables were performed with Pearson’s chi-squared test or Fisher´s exact test. Linear regression models for group differences adjusted by age for were fitted for the analysed prognostic scales. Cumulative annual incidence was calculated considering the number of deaths and annual population as registered in the Spanish Statistical Office. A p-value less than 0.05 (typically ≤ 0.05) was considered statistically significant. Data was analysed using SPSS V24.

The study followed the principles of the Declaration of Helsinki, and the protocol was approved by the Navarre´s Clinical Research Ethics Committee. The study uses data from the Major Trauma Registry of Navarra. This registry is an institutional database created in 2010 by the Navarra Health Department (ORDEN FORAL 53/2010, [https://bon.navarra.es/es/anuncio/-/texto/2010/79/16]).

The use of this database for clinical research purposes has been approved by the Navarre´s Clinical Research Ethics Committee (protocol 2017 with ethical statement number: Pyto 2017/92 and protocol 2021 with ethical statement number: PI_2021/126) and by the Data Protection Officer of Navarrabiomed Biomedical Research Center. Additionally, it specifies that the project complies with GCP standards (CPMP/ICH/135/95).

The data is recorded on the platform anonymized from the origin, which means that the personal data protection regulations do not apply to these data according to both committees and because of that the informed consent was waived by the IRB (Navarre´s Clinical Research Ethics Committee).

## Results

From the 2,610 patients included over a 10-year study period, 1,547 (59.3%) survived and 1,063 (40.7%) died. From the latter group, 624 (23.9% of all study patients) died out-of-hospital and 439 (16.8% of all study patients) in-hospital.

**Fig. 1:**
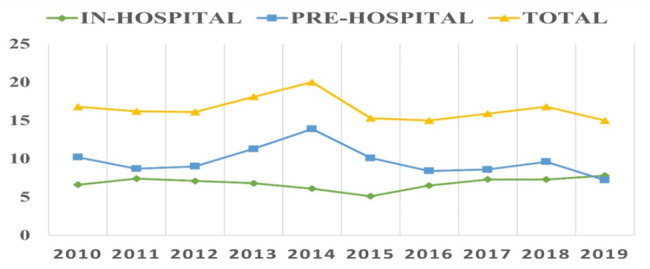
Number of deaths per 100,000 population by groups, including all seriously injured patients

Trauma incidence remained moderately stable over the 10 years analysed, with a slight decrease in out-of-hospital deaths and a slight increase in in-hospital deaths. Peak incidence of OOHDs in 2014 is worth noting, which later decreased and remained stable. (Fig. 1).

**Fig. 2:**
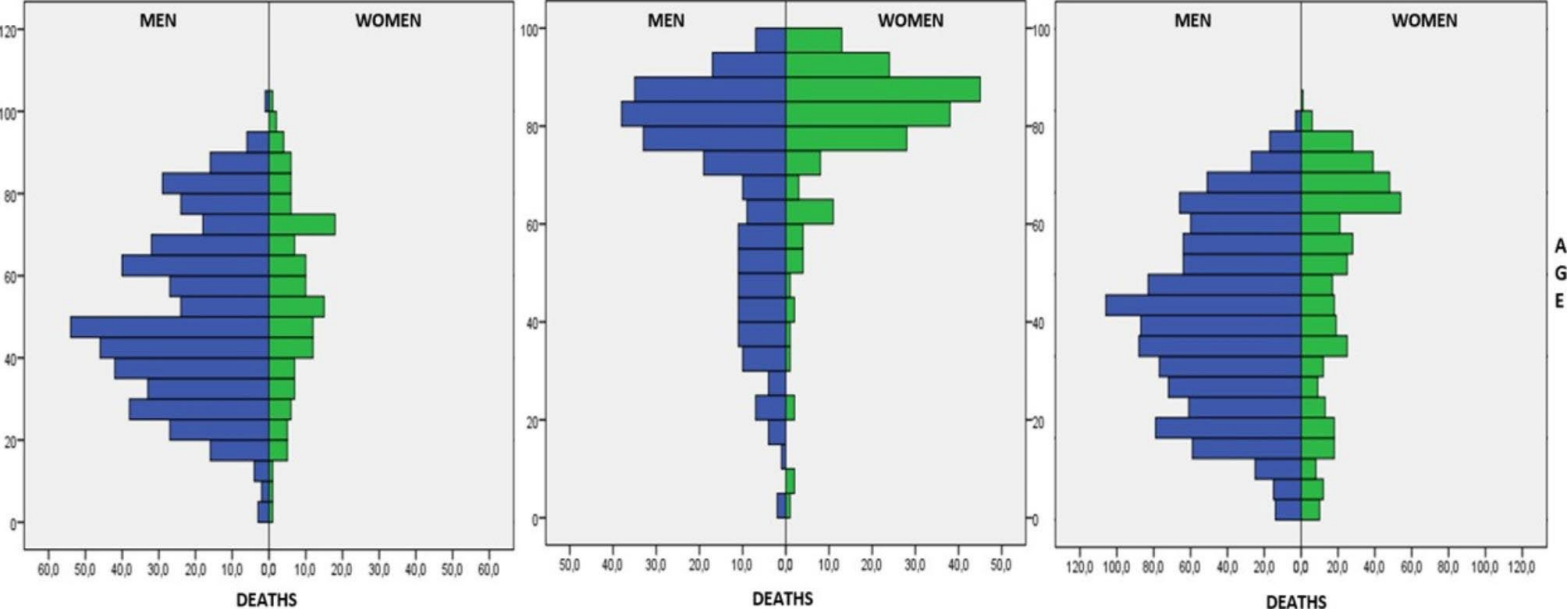
Deaths by age and sex. (**A**) Out-of-hospital deaths; (**B**) In-hospital deaths; (**C**) Survivors

The characteristics of the studied population are shown in Table 2. Patients who died out-of-hospital and survivors were younger (mean age of 50.9 and 51.4 years, respectively) while for IHDs and survivors mean age was 72 years (p-value < 0.001). Among patients with major trauma, 70.9% were male and were majority in all study groups. In IHDs, results were more similar, that is 57.2% male and 42.8% female (Fig. 2).

Regarding prior comorbidities, in most OOHD cases no data were found. On the other hand, 54.2% of the patients who died in-hospital (IODs) had one mild systemic disease and 68.1% of the survivors were healthy. In all groups, injuries were primarily caused by blunt force or accidental intentionality. However, the OOHD group showed the highest proportion of penetrating injuries (11.4%) and self-inflicted ones (34.0%). As for the mechanisms of OOHDs, traffic accidents (car, bus, and truck) led the list (26.9%), followed by fall from high heights (25%). On the contrary, in IHDs and survivors the leading cause was fall from low heights (57.6% and 31%, respectively) and in 26.4% of IHD the INR was higher than 2. Regarding airway handling, in 88.3% of the cases, no invasive methods were used; in 11.7% of the patients, some type of procedure was performed, such as the placement of an endotracheal tube or supraglottic airway.

**Table 2:** Characteristics of the study population

Variable	Category	Total	Out-of-hospital deaths (n = 624, 23.9%)	In-hospital deaths (n = 439, 16.8%)	Survivors (n = 1547, 59.3%)	p-value
**Age**	Mean (SD)		50.9 (20.8)	72.0 (20.6)	51.4 (23.5)	< 0.001^1^
**Sex**	Male	1851 (70.9%)	482 (77.2%)	251 (57.2%)	1118 (72.3%)	
	Female	759 (29.1%)	142 (22.8%)	188 (42.8%)	429 (27.7%)	< 0.001^2^
**Prior comorbidities / ASA-PS**	Unknown	641 (24.6%)	604 (96.8%)	6 (1.4%)	31 (2.0%)	
	Healthy	1204 (46.1%)	19 (3.0%)	132 (30.1%)	1053 (68.1%)	
	Mild systemic disease	658 (25.2%)	1 (0.2%)	238 (54.2%)	419 (27.1%)	< 0.001
	Serious systemic disease	107 (4.1%)	0 (0.0%)	63 (14.4%)	44 (2.8%)	
**Predominant type of trauma**	Penetrating	139 (5.3%)	71 (11.4%)	9 (2.1%)	59 (3.8%)	
	Blunt	2471 (94.7%)	553 (88.6%)	430 (97.9%)	1488 (96.2%)	< 0.001^2^
**Intentionality**	Accidental	2246 (86.1%)	389 (62.3%)	408 (92.9%)	1449 (93.7%)	
	Self-inflicted	291 (11.1%)	212 (34.0%)	16 (3.6%)	63 (4.1%)	
	Aggression/other	73 (2.8%)	23 (3.7%)	15 (3.4%)	35 (2.3%)	< 0.001^3^
**Mechanism**	Traffic (car, bus, truck)	479 (18.4%)	168 (26.9%)	38 (8.7%)	273 (17.6%)	
	Traffic (motorbike)	179 (6.9%)	29 (4.6%)	15 (3.4%)	135 (8.7%)	
	Traffic (bicycle)	141 (5.4%)	8 (1.3%)	9 (2.1%)	124 (8.0%)	
	Traffic (run over)	176 (6.7%)	40 (6.4%)	43 (9.8%)	93 (6.0%)	
	Firearm	59 (2.3%)	49 (7.9%)	2 (0.5%)	8 (0.5%)	
	Stabbing	42 (1.6%)	18 (2.9%)	4 (0.9%)	20 (1.3%)	< 0.001^2^
	Contusion by diverse objects	151 (5.8%)	37 (5.9%)	15 (3.4%)	99 (6.4%)	
	Fall from low height	775 (29.7%)	43 (6.9%)	253 (57.6%)	479 (31.0%)	
	Fall from high height	438 (16.8%)	156 (25.0%)	52 (11.8%)	230 (14.9%)	
	Other	170 (6.5%)	76 (12.2%)	8 (1.8%)	86 (5.6%)	
**Intubation**	No	1800 (88.3%)	40 (50.6%)	326 (76.3%)	1434 (93.6%)	
	Yes	238 (11.7%)	39 (49.4%)	101 (23.7%)	98 (6.4%)	< 0.001^2^
Oxygen therapy	No	840 (41.9%)	30 (40.0%)	174 (41.2%)	636 (42.1%)	
	Yes	1166 (51.6%)	45 (60.0%)	248 (58.8%)	873 (57.9%)	0.893^2^
Fluid therapy	No	798 (39.7%)	31 (41.3%)	164 (38.9%)	603 (39.8%)	
	Yes	1213 (60.3%)	44 (58.7%)	258 (61.1%)	911 (60.2%)	0.897^2^

^1^ Kruskal-Wallis test ^2^ Chi-Squared test ^3^ Fisher’s exact test

Table 3 shows the results of a multinomial logistic regression model to study the factors associated with the mortality groups. Compared to the OOHD group, patients in IHD group were older (OR 1.03 95% CI 1.02–1.05), with less self inflicted injuries (OR 0.19 95% CI 0.07–0.51), greater presence of falls from low height (OR 13.7 95% CI 4.84–38.8), and more fluid therapy administration (OR 3.47 95% CI 1.39–8.66). Regarding the survivors, compared to the OOHD group, they were less likely to present self-inflicted or aggression injuries, with a greater presence of falls from low height (OR 8.71 95% CI 3.16, 24.0).

**Table 3:** Multinomial logistic regression model comparing the mortality groups

Variable	Category	In-hospital deaths	p-value	Survivors	p-value
**Age**		1.03 (1.02, 1.05)	< 0.001	0.99 (0.98, 1.00)	0.077
**Sex**	Male	ref			
	Female	1.22 (0.66, 2.26)	0.529	1.08 (0.60, 1.92)	0.806
**Predominant type of trauma**	Penetrating	ref			
	Blunt	5.02 (0.56, 45.2)	0.151	2.92 (0.55, 15.6)	0.211
**Intentionality**	Accidental	ref			
	Self-inflicted	0.19 (0.07, 0.51)	0.001	0.13 (0.06, 0.31)	< 0.001
	Aggression/other	1.41 (0.38, 5.23)	0.607	0.20 (0.06, 0.64)	0.006
**Mechanism**	Traffic	ref			
	Arm	1.99 (0.14, 29.21)	0.614	1.90 (0.24, 15.0)	0.541
	Fall from low height	13.7 (4.84, 38.8)	< 0.001	8.71 (3.16, 24.0)	< 0.001
	Fall from high height	1.60 (0.70, 3.69)	0.266	1.38 (0.65, 2.93)	0.403
	Other	1.71 (0.54, 5.41)	0.361	2.51 (0.90, 7.01)	0.080
**Oxygen therapy**	No	ref			
	Yes	1.28 (0.51, 3.19)	0.596	0.87 (0.38, 2.01)	0.743
**Fluid therapy**	No	ref			
	Yes	3.47 (1.39, 8.66)	0.008	2.09 (0.90, 4.83)	0.085

Risk assessment scales used in patients with major trauma are shown in Table 4. Mean results for forensic ISS and NISS were higher in OOHDs in comparison to IHDs (ISS of 49.5 vs. 33.5 and NISS of 54.2% vs. 39%, respectively). On the contrary, ISS and NISS in survivors were lower in comparison to the other two study groups. Forensic ISS and NISS in OOHDs were higher than IHDs. Mean RTS was 0.2 (SD = 0.5) for OOHDs, 6.3 (SD = 1.8) for IHDs, and 7.5 (SD = 0.8) in survivors. However, it should be emphasised that in many of the registries the respiratory rate at triage was unavailable, hindering the calculation of the RTS. Clear differences were also observed in TRISS injury severity score. After adjusting for age, all the observed between-group differences were maintained.

**Table 4:** Prognostic scales

Variable	Out-of-hospital deaths (n = 624, 23.9%)	In-hospital deaths (n = 439, 16.8%)	Survivors (n = 1,547, 59.3%)	p-value	Beta (95%CI) *	p-value*
**Forensic ISS**	49.5 (23.0)	29.7 (13.3)	-	< 0.001^1^	17.1 (14.3, 19.8)	< 0.001
**Hospital ISS**	-	25.7 (11.3)	18.6 (7.5)	< 0.001^1^	9.0 (8.1, 10.0)	< 0.001
**Forensic NISS**	54.2 (20.6)	39.9 (19.4)	-	< 0.001^1^	11.9 (9.3, 14.5)	< 0.001
**Hospital NISS**	-	34.2 (12.8)	25.5 (8.2)	< 0.001^1^	10.9 (9.8, 11.9)	< 0.001
**RTS**	0.2 (0.5)	6.3 (1.8)	7.5 (0.8)	< 0.001^2^	-7.4 (-7-4, -7.3) ^3^ -1.4 (-1.5,-1.3)^4^	< 0.001
**TRISS**	0.03 (0.02–0.03)	0.69 (0.66, 0.71)	0.93 (0.93, 0.94)	< 0.001^2^	-0.91 (-0.92, -0.89)^3^ -0.25 (-0.27, -0.24)^4^	< 0.001

^1^ Student’s t-test ^2^ Kruskal-Wallis test; ^3^ Out-of-hospital deaths vs. survivors ^4^ In-hospital deaths vs. survivors *Adjusted by age

## Discussion

This study includes all registered cases of major trauma in Navarre between 2010 and 2019, with no major changes in the incidence of trauma during this period More than half of the registered deaths occur out-of-hospital and we observe significant differences between patients who die outside the hospital, those who die in the hospital and survivors in demographic variables such as sex and age, in injury related variables such as mechanism and intentionality [18], and in patient prognostic scales such as RTS and TRISS.

The three mortality groups described above show notable differences, In the IHD group, patients were 20 years older than in the OOHD and survivor groups, with a greater proportion of women and with a greater presence of comorbidities compared with the survivors. In the OOHD group, the proportion of self-inflicted injuries was significantly higher than in the IHD and survivors groups, in which the majority of injuries were accidental. Traffic injuries and falls from high height were the main risk factors in order to die out of the hospital, while the mechanism most associated with in-hospital deaths was fall from low height.

In the OOHD group, due to the severity of the injuries or the immediacy of death, a large proportion of these cases were not treatable by our emergency systems at the time of the event, therefore their reduction would happen almost exclusively through the prevention. Campaigns to prevent traffic accidents, drug abuse or improve mental health care could be effective, because of the high proportion of accidents and self-inflicted injuries. Mental disorders and drug abuse, not considered in our database, are prevalent in this group and should be considered, as risk factors [19–21] along with some of the traffic accidents classified as accidental may have been self-inflicted [22]. On the other hand, the prevention of deaths in IHD group goes through regarding the infra-triage and specific management of this entity considering the frailty of this patients and the usual polypharmacy in them [1, 23–25]. Emphasis should be placed on the proper use of benzodiazepines (in order to prevent falls) and anticoagulants due to the presence of an important percentage of patients with INR greater than 2, especially relevant as possible causes of cerebral haemorrhages with fatal outcome.

The ISS, NISS, and RTS classify injuries based on their severity or physiological effect. The higher the ISS and NISS or the lower the RTS, the more severe the injury. After reviewing the cases, we observed that these scales categorized some patients who had greater survival expectations but died few days later. This led us to hypothesize that these scales can become good predictors of the need of intensive care rather than mortality. Hence, they should be employed as predictors for survival by scales such as the Trauma Injury Severity Score, the Modified Rapid Emergency Medicine Score, or the Revised Injury Severity Classification. Furthermore, the Shock Index (associated to age), the Glasgow Coma Score, and RETRASCORE have shown to be superior when it comes to predicting hospital mortality in comparison to other scales and should be included in our trauma registry [17, 26–31].

The data collected for our analysis indicated that most traumas were blunt force injuries (94.7%), similar to the results obtained in other articles [1]. These figures only switch in favour of penetrating trauma [1] in conflict areas or urban epidemic zones in the USA (20–45%) or South Africa (up to 60%) [1].

As to survivors, firstly, it would be advisable to monitor long-term impairment caused by major trauma by using scales specifically developed for this purpose, such as the Quality of Well Being scale or the Health Utilities Index [1, 32]. Secondly we should pursue, the effective implementation of adequate social and economic resources aimed at vulnerable populations like the elderly and patients with mental health disorders; in the latter cases, suicide prevention strategies at national level should be introduced, as the National Strategy for Suicide Prevention in the USA [33–35].

Mortality in patients with severe trauma has traditionally been linked to a quarterly distribution model according to various sources, including concepts such as the golden hour. This model has been submitted on several occasions to trial without being able to clarify the doubts that have arisen in this regard, to remain valid today or not [36]–[37]. Regarding the golden hour, in our study we observed some difficulty in determining the factors that generate death in these patients and it cannot be ruled out that several of them converge at the same point in time, such as, the treatment received in the first 60 min, the type of injury, age and anatomical location affected. Therefore, more studies are needed to determine the individual magnitude of each factor separated from other variables. It is also essential to improve prevention mechanisms to increase efficacy and efficiency [38]. However, although in our study we did not identify cases whose survivability prospects would have improved with immediate emergency care, some authors maintain that in cases of severe head trauma, the prognosis improves when airway management is performed in time [39]–[40].

There are limitations to this study. Its observational design conditions the evidence for most diagnostic and therapeutic strategies applied to polytrauma patients, as it does not allow to infer causality but to link variables. However, we think that in some cases these links are consistent enough to propose preventive actions and limit the consequences, as in the case of falls in the elderly or the significant difference of OOHDs due to self-inflicted injuries, in comparison to the other study groups. In the latter two examples, the main precipitating factors are well known (anticoagulation in the elderly and mental disorders, respectively). Thus, early monitoring in these groups should be carried out with the appropriate resources.

In conclusion, there are differences in the characteristics of the patients between the three groups, OOHD, IHD and survival. In the OOHD group, the majority are young patients who die in traffic accidents and falls from great heights, while in the IHD group, falls from low heights stand out in elderly patients. In the case of survivors, young patients with falls from a low height are the most prominent.

Therefore, prevention is essential in the areas of transport and the development of geriatric strategies, as well as areas with increasing relevance such as mental health and drug use in patients in the OOHD group and frailty in the IHD group. Therefore, the preventive aspect is vital, since certain injuries that lead to death are difficult to resolve in serious trauma once they are established.

The accomplishment of this study was possible due to research aid PI17/00645. Sponsored as a Health Research Project 2017 of the Health Strategic Action. FIS 2018–2020.

## Data Availability

The datasets used and/or analysed during the current study are available from the corresponding author on reasonable request.

## List of Abbreviations

NISS: New Injury Severity Score
OOHDs: Out-of-hospital deaths
IHDs: In-hospital deaths
ISS: Injury Severity Score
RTS: Revised Trauma Score
TRISS: Trauma Injury Severity Score
ASA-PS: The American Society of Anaesthesiologists physical status
